# Radiological characteristics and predisposing factors of venous tumor thrombus in pelvic osteosarcoma: A mono‐institutional retrospective study of 115 cases

**DOI:** 10.1002/cam4.1739

**Published:** 2018-08-23

**Authors:** Haijie Liang, Wei Guo, Rongli Yang, Xiaodong Tang, Taiqiang Yan, Tao Ji, Yi Yang, Dasen Li, Lu Xie, Jie Xu

**Affiliations:** ^1^ Musculoskeletal Tumor Center Peking University People's Hospital Beijing China

**Keywords:** chondroblastic subtype, intervertebral foramen, osteosarcoma, pelvis, venous tumor thrombus

## Abstract

**Background:**

Venous tumor thrombus (VTT) in pelvic osteosarcoma has been regarded as a rare oncological condition and few literatures investigated this issue.

**Methods:**

We retrospectively reviewed 115 cases of pelvic osteosarcoma treated in our center from 2006 to 2016. Diagnosis of VTTs was made based on histo‐pathological findings. We summarized the radiological manifestations of VTTs on CT, MRI, and PET/CT. We also compared the demographical, oncological, and radiological data between cases with or without VTTs to identify its predisposing factors.

**Results:**

Seventeen cases (14.8%) were diagnosed with VTT. Manifestations of VTTs on CT included increased caliber (64.7%), calcification (47.1%), low density on plain scan (100%), filling defect (100%), and streak‐like enhancement (35.7%) on contrast enhancement. On MRI, the VTTs could be hypo‐ or iso‐intense on T1WI (100%), hyperintense on T2WI (100%), and filling defect on contrast enhancement (100%). PET/CT showed high metabolic activity of the VTTs. The ranges of the VTTs included unilateral external iliac vein (EIV) (two cases), unilateral internal iliac vein (IIV) (one case), unilateral common iliac vein (CIV) and IIV (five cases), unilateral CIV + EIV (two cases), inferior vena cava (IVC) and unilateral CIV + EIV (one case), IVC and unilateral CIV + IIV (four cases), IVC and bilateral CIVs + IIVs (two cases). Multivariate analysis indicated that chondroblastic subtype and involvement of L5/S1 intervertebral foramen might predispose to VTTs.

**Conclusion:**

The incidence of VTTs in pelvic osteosarcoma was 14.8%. Comprehensive radiological studies help preoperative diagnosis of VTTs. Predisposing factors included chondroblasic subtype and involvement of L5/S1 intervertebral foramen.

## 1 INTRODUCTION

Venous tumor thrombus (VTT) is not uncommonly seen in some retroperitoneal malignancies such as renal cell carcinoma^1^ and is occasionally noted as a rare condition in patients with osteosarcoma according to the previous literature.^1^, ^2^, ^3^, ^4^, ^5^, ^6^, ^7^, ^8^, ^9^, ^10^, ^11^, ^12^, ^13^, ^14^, ^15^, ^16^, ^17^ Almost all of the literal records about VTT in osteosarcoma were case reports with extremely poor outcomes and the pelvic ring was the most common location of those cases.^1^, ^4^, ^6^, ^7^, ^8^, ^9^, ^11^, ^13^

Pelvic osteosarcomas accounted for only <10% of all osteosarcomas and their outcomes remained disappointing despite aggressive chemotherapy and surgery, with 5‐year overall survival (OS) ranging from 18% to 45%.^4^, ^18^, ^19^, ^20^, ^21^, ^22^, ^23^, ^24^, ^25^, ^26^, ^27^, ^28^, ^29^, ^30^, ^31^ The outcome would be much worse when a pelvic osteosarcoma was complicated by VTT and then a treatment dilemma would be encountered.^1^, ^4^, ^6^, ^7^, ^8^, ^9^, ^11^, ^13^ Moreover, an unanticipated VTT found during operation would tremendously increase the risk of perioperative mortality and repudiate the significance of invasive resection of the tumor due to the compromised margin within the vessel.^1^, ^6^, ^7^, ^8^, ^9^, ^11^ As a result, it is necessary to investigate on VTT in pelvic osteosarcomas and figure out its incidence, radiological manifestations, and predisposing factors in order to help practitioners to identify and manage this oncological condition.

## 2 MATERIALS AND METHODS

### 2.1 Data collection

This study was approved by the institutional review board. Informed consents were obtained from all subjects or guardians. We retrospectively reviewed the charts of patients with pelvic osteosarcomas treated in our center from January 2006 to December 2016. Cases of secondary osteosarcomas, or cases treated nonsurgically, were excluded. Eventually 115 cases were included in this study. Demographic data, oncological parameters, and pathological diagnosis, laboratory results, radiological manifestations of the tumor at presentation, and oncological outcomes were explicitly reviewed and documented. Diagnoses of VTTs were confirmed in 17 cases by intra‐operative exploration and histo‐pathological examination, and their radiological characteristics were summarized.

### 2.2 Statistical analysis

Univariate analysis of the correlation between the variables and the VTT was performed by the chi‐square test or Fisher's exact test for category variables and by the Student *t* test for the continuous variables. Variables with a probability value (*P*) <0.1 in univariate analysis were entered in a binary‐logistic regression model for multivariate analysis. The oncological survival was analyzed by Kaplan‐Meier curves and log‐rank testing. A *P* value of less than 0.05 was considered to be significant. Statistical analysis was performed using the Statistical Package for the Social Science (SPSS) software version 23.0 (SPSS Inc, Chicago, IL, USA).

## 3 RESULTS

### 3.1 Demographic data of the patients

There were 63 males and 52 females in this cohort with a mean age of 31.0 ± 14.8 years (mean ± SD). The mean duration from symptom onset to presentation was 9.2 ± 14.9 months. Ninety‐three patients (80.9%) sought for primary treatments, while the rest patients were previously treated elsewhere and presented with recurrence. The Enneking stages for this cohort included 5 (4.3%) cases of IIA, 83 (72.2%) cases of IIB, and 27 (23.5%) cases of III. Of those cases with metastatic diseases, there were 24 cases with lung metastasis, three cases with lymph node metastasis, and three cases with skeletal metastasis. According to Enneking and Dunham's classification of pelvic lesions,^32^ there were six cases of type I, one case of type II, one case of type III, 18 case of type II+III with three of them involving proximal femur, 16 cases of type I+II with one of them involving proximal femur, 17 cases of type I+II+III with two of them involving proximal femur, 23 cases of type I+IV, 17 cases of type I+II+IV, 16 cases of type I+II+III+IV with one of them involving proximal femur. The averaged greatest diameter of the tumor was 112.7 ± 36.6 mm (Table 1).

**Table 1:** Baseline data of 115 cases of pelvic osteosarcoma

Variables	Value
Gender [N (%)]	
Male	63 (54.8%)
Female	52 (45.2%)
Age (yr, mean ± SD)	31.0 ± 14.8
Height (cm, mean ± SD)	168.6 ± 8.8
Weight (kg, mean ± SD)	61.3 ± 13.5
BMI (kg/m^2,^ mean ± SD)	21.5 ± 3.9
Onset duration (mo, mean ± SD)	9.2 ± 14.9
Histological subtype [N (%)]	
Chondroblastic	34 (29.6%)
Well‐differentiated with dedifferentiation	11 (9.6%)
Osteoblastic	19 (16.5%)
Fibroblastic	11 (9.6%)
Telangiectatic	2 (1.7%)
Small cell	6 (5.2%)
Epithelioid	1 (0.9%)
Not otherwise specified	31 (27.0%)
Enneking staging for primary tumor [N(%)]	
IIA	5 (4.3%)
IIB	83 (72.2%)
III	27 (23.5%)
Classification of pelvic tumor [N(%)]	
Type I	6 (5.2%)
Type II	1 (0.9%)
Type III	1 (0.9%)
Type II+III	15 (13%)
Type II+III+proximal femur	3 (2.6%)
Type I+II	15 (13%)
Type I+II+proximal femur	1 (0.9%)
Type I+II+III	15 (13%)
Type I+II+III+poximal femur	2 (1.7%)
Type I+IV	23 (20%)
Type I+II+IV	17 (14.8%)
Type I+II+III+IV	15 (13%)
Type I+II+III+IV+proximal femur	1 (0.9%)
Greatest diameter of the tumor (mm, mean ± SD)	112.7 ± 36.6
Metastasis at presentation [N (%)]	
Lung	24 (20.9%)
Lymph node	5 (4.3%)
Other sites	3 (2.6%)
Venous tumor thrombus at presentation [N (%)]	17 (14.8%)

### 3.2 Radiological characteristics of venous tumor thrombus (VTT)

There were 17 cases (14.8%) presenting with VTT that could be identified by radiological examinations. The VTTs were located in unilateral external iliac vein (EIV) in two cases (11.8%), in unilateral internal iliac vein (IIV) in one case (5.9%), in unilateral common iliac vein (CIV) + IIV in five cases (29.4%), in unilateral CIV+EIV+IIV in two cases (11.8%), in inferior vena cava (IVC) + unilateral CIV + EIV in one case (5.9%), in IVC+ unilateral CIV+IIV in four cases (23.6%), in IVC+ bilateral CIV+IIV in two cases (11.8%). There was a tendency that the internal iliac vein would be involved in these cases (82.3%; Table 2).

**Table 2:** Ranges of the venous tumor thrombi in 17 cases

Ranges	N (%)	Classification of pelvic tumor
Unilateral external iliac vein	2 (11.8%)	Type I+II+III *1
		Type I+II+IV *1
Unilateral internal iliac vein	1 (5.9%)	Type I+II+IV *1
Unilateral common and internal iliac veins	5 (29.4%)	Type I+IV *2
		Type I+II+IV *2
		Type I+II+III+IV*1
Unilateral common, internal and external iliac veins	2 (11.8%)	Type II+III *1
		Type I+II+III+IV*1
Inferior vena cava, unilateral common and external iliac veins	1 (5.9%)	Type II+III *1
Inferior vena cava, unilateral common and internal iliac veins	4 (23.6%)	Type 1 *1
		Type I+IV *1
		Type I+II+III+IV *2
Inferior vena cava, bilateral common and internal iliac veins	2 (11.8%)	Type I+IV *1
		Type I+II+III *1

**Figure 1:**
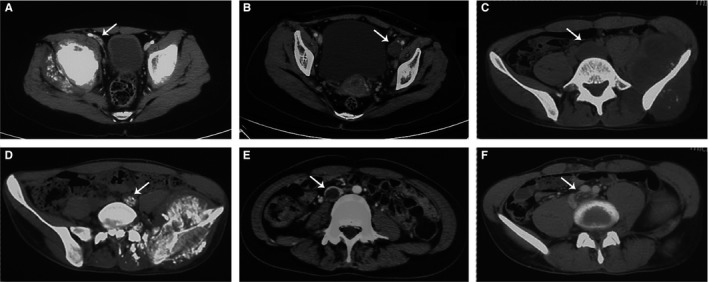
Manifestations of venous tumor thrombus (VTT) on plain and enhanced CT scan. A, A VTT located at the right external iliac vein (arrow) showed unchanged caliber of the vessel compared with the contralateral side. B, A VTT located at the left external iliac vein (arrow) showed enlarged caliber of the vessel compared with the contralateral side. C, A VTT located at the left common iliac vein (arrow) showed low density compared with the muscles on plain CT scan. D, A VTT located at the left common iliac vein (arrow) showed apparent calcification within the vascular lumen on plain CT scan. E, A VTT located at the inferior vena cava (arrow) showed filling defect within the vascular lumen on contrast enhancement. F, A VTT located at the left common iliac vein (arrow) showed streak‐like enhancement within the filling defect on contrast enhancement

The manifestations of VTT under enhanced computed tomography (CT) scan were as follows (Figure 1). The caliber of the vein could be unchanged (six cases, 35.3%) or enlarged (11 cases, 64.7%). All of the VTTs were of low density and eight of them showed segmented calcification within the lumen under plain scan. On contrast enhancement, filling defect within the lumen was seen in all cases with five of them (29.4%) showing linear enhancement inside the thrombus (ie, thread and streak sign).

**Figure 2:**
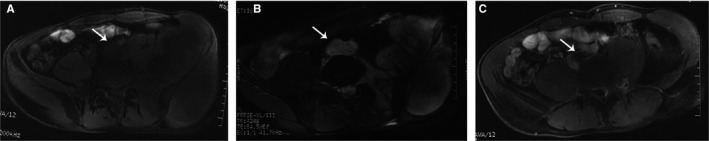
Manifestations of venous tumor thrombus (VTT) on plain and enhanced MRI scan. A, A VTT located at the left common iliac vein (arrow) showed hypointense fillings inside the vascular lumen on T1WI sequence. B, A VTT located at the left common iliac vein (arrow) showed hyperintense fillings inside the vascular lumen on T2WI sequence, which was apparent compared with the flowing void effect of the right common iliac vein. C, A VTT located at the left common iliac vein (arrow) showed streak‐like enhancement within the filling defect on contrast enhancement

^18^FDG positron emission tomography/computed tomography (PET/CT) was performed in 3 of the 17 cases (Figure 3), all of which showed high ^18^FDG uptake within range of the VTTs (Table 3).

Magnetic resonance imaging (MRI) was performed in 14 of the 17 cases (Figure 2). Under plain scan, all of the VTTs (14/14, 100%) manifested as fillings within the involved veins with hypo‐ or iso‐intensity in T1WI and hyperintense in T2WI sequences, which was noticeable compared with the flow‐void phenomenon of the other normal veins. Filling defect within the lumen was also seen in all cases under enhanced scan.

**Figure 3:**
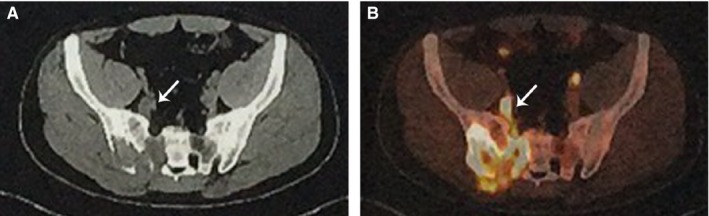
Manifestations of venous tumor thrombus (VTT) on PET/CT scan. A, Plain CT scan showed an osteosarcoma located at the right sacroiliac joint with a slightly enlarged internal iliac vein with low dense fillings (arrow), indicating a VTT within the internal iliac vein. B, PET scan showed high metabolic activity at the right sacroiliac joint and at the site of right internal iliac vein (arrow)

**Table 3:** Radiological manifestations of the venous tumor thrombi in 17 cases

Radiological manifestations	N (%)
CT (N = 17)	
Unchanged caliber	6 (35.3%)
Enlarged caliber	11 (64.7%)
Calcification	8 (47.1%)
Low density on plain scan	17 (100%)
Filling defect on enhancement	17 (100%)
Streak‐like enhancement	5 (29.4%)
MRI (N = 14)	
Hypo‐ or iso‐intensity on T1WI	14 (100%)
Hyper‐intensity on T2WI	14 (100%)
Filling defect on enhancement	14 (100%)
Streak‐like enhancement	5 (35.7%)
PET/CT (N = 3)	
High metabolic activity	3 (100%)

### 3.3 Univariate analysis of the predisposing factors of VTT

Univariate analysis indicated that age (*P* = 0.015), BMI (*P* = 0.035), staging (*P* = 0.062), Involvement of sacrum (*P* = 0.05), chondroblastic subtype (*P* = 0.004), LDH level (*P* = 0.078), blockage of the greater sciatic foramen by the tumor (*P* = 0.052), invasion of the sacral foramen (*P* < 0.001), and invasion of L5/S1 intervertebral foramen (*P* < 0.001) might be related to the occurrence of VTT (Table 4).

**Table 4:** Univariate analysis of the potential predisposing factors of venous tumor thrombus

Variables	Non‐VTT group (N = 98)	VTT group (N = 17)	*P* value
Gender [N(%)]			
Male	53 (54.1%)	10 (58.8%)	0.717
Female	45 (45.9%)	7 (41.2%)	
Age (y, mean ± SD)	32.2 ± 15.0	23.9 ± 11.6	0.015
Height (cm, mean ± SD)	168.7 ± 8.5	168.0 ± 10.8	0.789
Weight (kg, mean ± SD)	62.2 ± 13.5	56.1 ± 12.4	0.079
BMI (kg/m^2^, mean ± SD)	21.7 ± 4.0	19.8 ± 3.2	0.035
Onset duration (mo, mean ± SD)	9.62 ± 15.6	7.0 ± 10.6	0.392
Primary presentation [N(%)]	77 (78.6%)	16 (94.1%)	0.188
Staging [N(%)]			
Localized	78 (79.6%)	10 (58.8%)	0.062
Metastatic	20 (20.4%)	7 (41.2%)	
Side of the lesion [N(%)]			
Left	52 (53.1%)	12 (70.6%)	0.179
Right	46 (46.9%)	5 (29.4%)	
Involvement of sacrum [N(%)]			
Yes	44 (44.9%)	12 (70.6%)	0.05
No	54 (55.1%)	5 (29.4%)	
Greatest diameter of the tumor (mm, mean ± SD)	110.7 ± 37.6	124.7 ± 28.2	0.166
Greatest diameter of the tumor [N(%)]			
<100 mm	34 (34.7%)	5 (29.4%)	0.671
≥100 mm	64 (65.3%)	12 (70.6%)	
Histological subtype [N(%)]			
Chondroblastic	24 (24.5%)	10 (58.8%)	0.004
Nonchondroblastic	74 (75.5%)	7 (41.2%)	
Initial laboratory test			
WBC (*10^9^/L)	6.42 ± 2.2	5.68 ± 1.7	0.206
Neutrophil (%)	61.3 ± 14.4	59.2 ± 16.9	0.608
Lymphocyte (%)	27.1 ± 11.5	29.9 ± 15.2	0.397
Hemoglobin (g/L)	123.3 ± 21.1	122.1 ± 18.5	0.822
Platelet (*10^9^/L)	249.0 ± 107.7	254.1 ± 65.8	0.856
ALP (U/L)	300.8 ± 449.7	377.5 ± 507.2	0.526
LDH (U/L)	268.3 ± 176.5	349.9 ± 160.9	0.078
PT (s)	11.4 ± 1.4	11.7 ± 1.8	0.511
APTT (s)	32.0 ± 3.4	33.3 ± 4.2	0.175
PTA (%)	93.7 ± 14.4	92.3 ± 16.8	0.718
Fibrinogen (mg/dL)	343.0 ± 106.9	327.9 ± 111.6	0.597
D‐dimmer (ng/mL)	733.5 ± 2140.6	338.8 ± 390.0	0.523
Blockage of the greater sciatic foramen [N(%)]			
Yes	18 (19.6%)	7 (41.2%)	0.052
No	74 (80.4%)	10 (58.8%)	
Invasion of the sacral foramen [N(%)]			
Yes	21 (21.4%)	11 (64.7%)	<0.001
No	77 (78.6%)	6 (35.3%)	
Invasion of L5/S1 intervertebral foramen [N(%)]			
Yes	4 (4.3%)	7 (41.2%)	<0.001
No	88 (95.7%)	10 (58.8%)	

VTT, venous tumor thrombus.

### 3.4 Multivariate analysis of the predisposing factors of VTT

Multivariate analysis including age, BMI, LDH level, metastatic stage, chondroblastic subtype, blockage of the greater sciatic foramen, Invasion of the sacral foramen, and invasion of L5/S1 intervertebral foramen showed that only chondrablastic subtype (OR: 4.964, *P* = 0.037) and invasion of L5/S1 intervertebral foramen (OR: 11.073, *P* = 0.013) remained as significant correlated factors of VTT (Table 5).

**Table 5:** Multivariate analysis of the predisposing factors of venous tumor thrombus

Variables	OR [95% CI]	*P* value
Age	1.007 [0.944, 1.075]	0.822
BMI	0.839 [0.675, 1.041]	0.111
LDH	1.000 [0.997, 1.004]	0.774
Metastatic stage	1.916 [0.464, 7.905]	0.369
Chondroblastic subtype	4.964 [1.102, 22.367]	0.037
Blockage of the Greater sciatic foramen	0.888 [0.190, 4.147]	0.880
Invasion of the sacral foramen	2.541 [0.504, 12.819]	0.259
Invasion of L5/S1 intervertebral foramen	11.073 [1.668, 73.522]	0.013

### 3.5 Oncological outcomes of localized pelvic osteosarcoma

**Figure 4:**
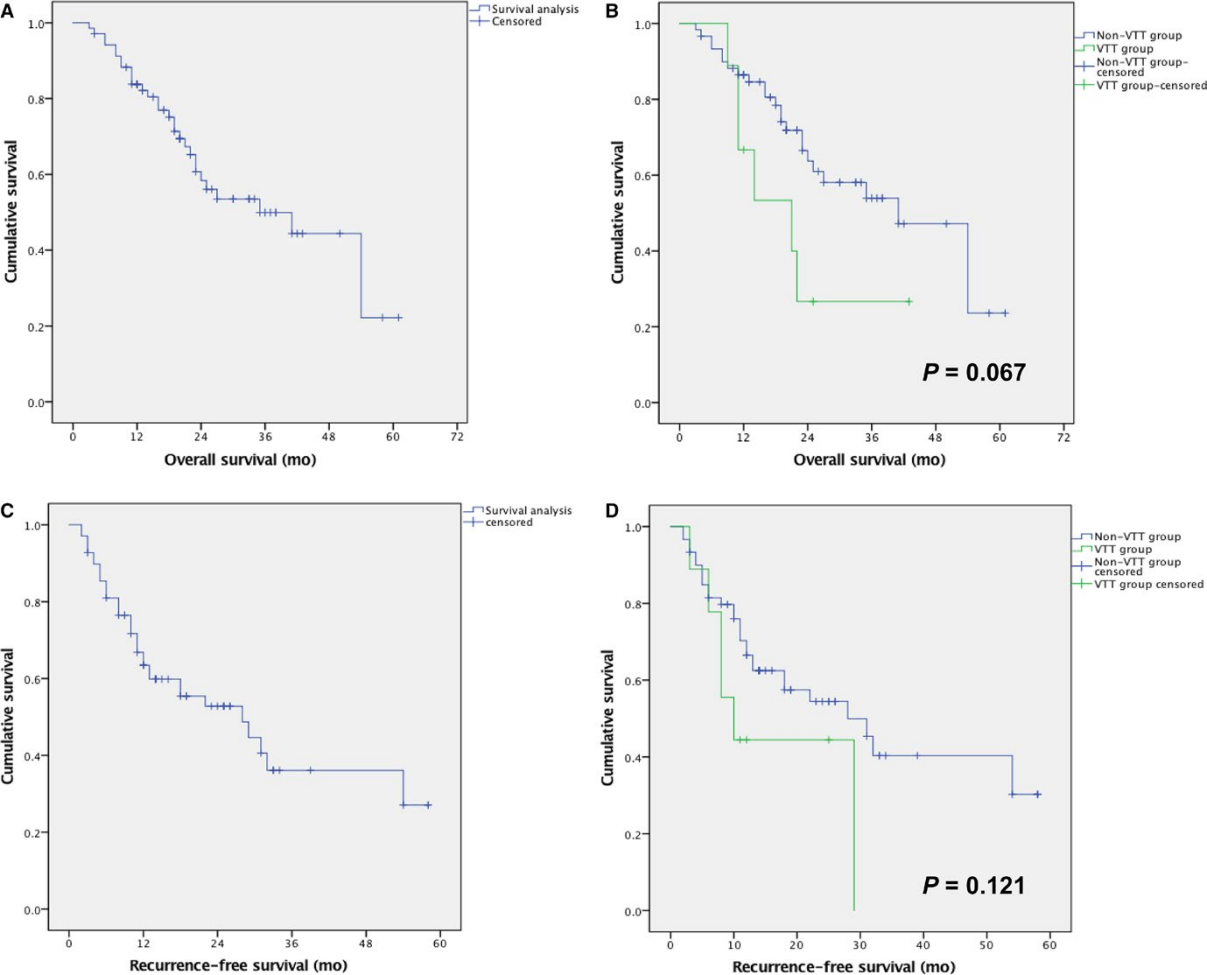
Kaplan‐Meier analysis for the overall survival and recurrence‐free survival for localized diseases of the whole cohort (A, C), as well as comparison between the non‐VTT and VTT groups (B, D)

The mean overall survival (OS) of the localized disease in this cohort was 36.5 ± 2.9 months. As for the non‐VTT group, the mean OS were 38.2 ± 3.2 months, while in the VTT group the result was 22.5 ± 4.6 months (*P* = 0.067, Figure 4B). In regard to recurrence‐free survival (RFS), the result was 30.0 ± 3.2 months for the whole cohort (Figure 4C). The mean RFS were 31.7 ± 3.4 months and 16.8 ± 4.0 months for the non‐VTT and VTT groups, respectively (*P* = 0.121, Figure 4D).

## 4 DISCUSSION

The VTT has been regarded as an insidious condition for pelvic osteosarcoma for a long time because there were few literatures investigating this topic. In this retrospective study based on 115 cases of pelvic osteosarcoma, we found that the incidence of VTTs in pelvic osteosarcoma was 14.8%, and that radiological examinations such as CT, MRI, and PET/CT could help identify the VTTs preoperatively, and that chondroblastic subtype and invasion of L5/S1 intervertebral foramen were correlated with the occurrence of VTTs.

### 4.1 Incidence of VTT in patients with pelvic osteosarcoma

Previous literatures about VTT in cases of osteosarcoma were majorly case reports, and thus, it was difficult to estimate the incidence of VTT based on them. Fahey et al^33^ once reported the presence of the tumor within the lumen of the large veins discovered intraoperatively without anticipation in 9 of 18 patients (50%) with pelvic osteosarcoma that received surgical treatments. Kawai et al^4^ reported that among the 30 patients with pelvic osteosarcoma receiving surgical resection, macroscopic tumor emboli within large veins were found in six cases,resulting the incidence of VTT as 20%. Similarly, our study demonstrated the incidence as 14.8% in a larger cohort of 115 cases, which was lower than those of previous reports but still high enough to raise the attention of clinical practitioners. Moreover, we speculated that the real incidence of VTT in pelvic osteosarcoma could be higher because we only included cases undergone surgeries in this study. Those nonsurgically treated cases were generally more advanced and might have a higher chance to develop VTTs.^1^, ^4^, ^6^, ^7^, ^8^, ^9^, ^11^, ^13^, ^14^

### 4.2 Radiological manifestations of VTT

It has been reported that CT and MRI scan with contrast, as well as^18^F‐FDG PET/CT could be used to detect the existence of VTTs in cases of osteosarcoma.^1^, ^6^, ^10^, ^11^, ^13^, ^14^ Based on this study, we highlighted that typical radiological manifestations of VTTs included increased caliber, low density with/without calcification within the lumen under plain CT scan, fillings of hypo‐ or iso‐intensity on T1WI and hyperintensity on T2WI under plain MRI scan, filling defect with/without streak‐like enhancement within the thrombus under enhanced CT or MRI scan. Clinicians and radiologists should be alerted to these signs and identify potential VTT promptly. However, the differentiation between VTT and nontumor thrombus could be difficult sometimes. According to our experience and previous literatures, the thrombus that shows calcification, streak‐like enhancement, homogenously hyperintensity on T2WI, FDG uptake in PET/CT, and normal serum level of D‐dimmer is more likely to be a tumor thrombus.^1^, ^2^, ^6^, ^10^, ^13^

### 4.3 Growing patterns of VTT

The extension range of the VTT reflected the growing pattern of tumor thrombus. Firstly, the origin of the VTT was closely related to the location of the tumor. For instance, a type I+IV pelvic osteosarcoma could have a VTT growing from branches of the internal iliac vein but never from external iliac vein, while a type II+III osteosarcoma could have a VTT growing from either the external or internal iliac vein. Secondarily, most of the VTTs involved the internal iliac vein (14 of 17 cases). This accorded with the normal venous draining of the pelvis as the internal iliac vein dominantly draining the blood outflow of the components of the pelvis (ie, bones, muscles and visceral organs). Thirdly, all of the VTTs that involved venous trunks (ie, common iliac vein or IVC) also had external or internal iliac vein invaded, indicating that the VTTs originated from peripheral venous branches of the tumor and then extended proximally to the venous trunks. Once the venous trunk was occluded, the tumor thrombus might keep growing both proximally and distally.

### 4.4 Predisposing factors of VTT

One would hold a concept that the bigger a tumor is, the more likely it would develop a VTT. However, it was not true according to our results (Table 4). Another natural perspective is that the longer interval of symptom onset before diagnosis, the longer time for the tumor to extend and develop tumor thrombus. This was also not true because our results showed that the average intervals were 9.62 ± 15.6 and 7.0 ± 10.6 months for the non‐VTT and VTT groups, respectively (*P* = 0.392). Based on the growing pattern of the VTT in pelvic osteosarcoma, we initially assumed that the invasion of the greater sciatic foramen or invasion of the sacral foramen would at a higher risk of developing VTT, as it would be easier to invade the gluteal veins or presacral venous plexus respectively. Nevertheless, multivariate analysis did not support these assumptions and showed that only chondrablastic subtype and invasion of L5/S1 intervertebral foramen were independently correlated with the occurrence of VTT.

Chondroblastic osteosarcoma has been noted to account for a higher portion of osteosarcoma in the pelvis than in the extremities,^4^, ^33^ which was also suspected as one of the reasons for the poor outcome of pelvic osteosarcoma due to the poor response to chemotherapy,^4^, ^19^, ^23^, ^26^ In this study, chondroblastic subtype was also found to be an independent correlated factor of VTTs in pelvic osteosarcoma (OR: 4.964, *P* = 0.037). The explanation for this result remained unclear and we assumed that it could be related to its chemo‐resistant nature or its potential intrinsic tendency to form tumor thrombus.

**Figure 5:**
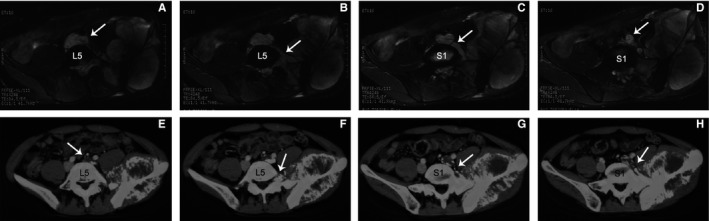
Typical cases of pelvic osteosarcoma with venous tumor thrombus (VTT) and involvement of the L5/S1 intervertebral foramen. A‐D, A 21‐year‐old male diagnosed as a type I+II+III+IV pelvic osteosarcoma of chondroblastic subtype. A, He was complicated by a VTT extending from left internal iliac vein to the left common iliac vein and the inferior vena cava (arrow). B, Axial MRI scans showed obvious involvement of the left L5/S1 intervertebral foramen (arrow). C‐D, A branch of the internal iliac vein filled with tumor thrombus surrounded the S1 vertebra linking the tumor with the common iliac vein (arrow). E‐H, A 26‐year‐old male diagnosed as a type I+II+III+IV pelvic osteosarcoma not otherwise specified. E, He was complicated by a VTT extending from left internal iliac vein to the left common iliac vein (arrow). F, Axial CT scans showed obvious involvement of the left L5/S1 intervertebral foramen (arrow). G‐H, A branch of the internal iliac vein filled with tumor thrombus surrounded the S1 vertebra linking the tumor with the common iliac vein (arrow)

Invasion of the L5/S1 intervertebral foramen was the other strong correlated factor of VTTs in this study (OR: 11.073, *P* = 0.013). It was easy to evaluate whether the tumor had extended to the L5/S1 intervertebral foramen by axial CT or MRI scanning (Figure 5). In those cases presenting with this sign and VTTs, there would always be a venous branch with tumor thrombus linking the lesion in the intervertebral foramen and the venous trunk, which was also filled with tumor thrombus (Figure 5). Based on this phenomenon, we speculated that the invasion of L5/S1 intervertebral foramen could be either a cause or a consequence of VTT. On the one hand, invasion of this site might provide access to the abundant perivertebral venous plexus so that the tumor could easily grow into the venous system from branches to the trunk. On the other hand, formation of VTT in the venous trunk might block the normal venous outflow direction and result in increased blood flow of the perivertebral venous plexus, which might facilitate the extension of the tumor to the L5/S1 intervertebral foramen. Further investigations, however, are required to figure out the relationship between this sign and formation of VTTs.

### 4.5 Outcomes, classification, and recommendations for management of VTT

The OS and RFS of localized disease in this cohort were 36.5 and 30 months, respectively, which were comparable to those of literatures.^4^, ^18^, ^19^, ^20^, ^21^, ^22^, ^23^, ^24^, ^25^, ^26^, ^27^, ^28^, ^29^, ^30^, ^31^ The results were much worse in the VTT group than those in the non‐VTT group (OS: 22.5 vs 38.2 months; RFS: 16.8 vs 31.7 months), indicating the detrimental effects of VTT.

To further investigate on this topic, we classified the patterns of VTTs into four types (Table 6). Type 1 VTTs refer to those involving unilateral internal (1a) or external iliac vein (1b). Type 2 VTTs refer to those involving unilateral common iliac vein plus internal iliac vein (2a), external iliac vein (2b), or both (2c). Type 3 VTTs refer to those involving inferior vena cava (below renal veins) and unilateral common iliac vein plus internal iliac vein (3a), external iliac vein (3b), or both (3c). Type 4 VTTs refer to those involving inferior vena cava (any level) plus bilateral iliac veins, or involving inferior vena cava above renal veins.

**Table 6:** Classification of venous tumor thrombus for pelvic osteosarcoma and proposed recommendations for management

Classification	Criteria	No. in this cohort	Management in this cohort	Outcome in this cohort		Recommendations
Type 1	Involvement of unilateral internal or external iliac vein					IVC filter insertion Resection (amputation) or complete thrombectomy (limb salvage)
1a	Unilateral internal iliac vein	1	1 Resection	LR & Met.	DOD at 7 mo	
1b	Unilateral external iliac vein	2	two complete thrombectomy	2 LR & Met.	1 AWD at 43 mo, 1 DOD at 6 mo	
Type 2	Involvement of unilateral common iliac vein + internal and/or external iliac vein					IVC filter insertion Resection (amputation) or complete thrombectomy (limb salvage)
2a	Unilateral common + internal iliac veins	5	one resection	Met.	AWD at 17 mo	
			one complete thrombectomy	LR & Met.	DOD at 21 mo	
			three partial thrombectomy^a^	1 loss of follow‐up; 1 Met.; 1 LR & Met.	2 DOD at 10, 11 mo respectively	
2b	Unilateral common + external iliac veins	0	/		/	
2c	Unilateral common + internal + external iliac veins	2	two partial thrombectomy	1 Met.; 1 LR & Met.	2 DOD at 12, 14 mo respectively	
Type 3	Involvement of IVC (below renal veins) + unilateral iliac veins					IVC filter insertion Nonsurgery, or complete thrombectomy in selected cases (very sensitive to neoadjuvant chemotherapy)
3a	IVC + unilateral common + internal iliac veins	4	one complete thrombectomy	NED	NED at 25 mo	
			three partial thrombectomy	1 NED; 2 LR & Met.	1 NED at 12 mo, 2 DOD at 9, 22 mo respectively	
3b	IVC + unilateral common + external iliac veins	1	Nonsurgery	LR & Met.	DOD at 11 mo	
3c	IVC + unilateral common + internal + external iliac veins	0	/		/	
Type 4	Involvement of IVC (any level) + bilateral iliac veins or involvement of IVC (above renal veins)	2	Nonsurgery	2 LR & Met.	DOD at 10, 13 mo respectively	IVC filter insertion Nonsurgery

IVC, inferior vena cava; LR, local recurrence; Met., metastasis; DOD, died of disease; AWD, alive with disease; NED, no evidence of disease.

a Partial thrombectomy was performed because part of the thrombus grew into the venous wall and could not be resected completely.

Management of the VTT requires carefully evaluation for the resectability of the thrombus and highly skillful surgical techniques. The influence of elimination of the VTT on local control or distant control remains unknown and would require further investigations. According to our limited experience, we proposed a strategy for management of the VTT based on the above classification (Table 6). Firstly, all of the patients with VTTs should receive IVC filter insertion in order to prevent acute pulmonary embolism. For type 1a VTT, the whole internal iliac vein could be resected with the tumor conveniently without serious consequences, while type 1b VTT would require complete thrombectomy and preserve the external iliac vein in limb‐salvage cases, or resection with the tumor in amputation cases. For type 2 cases, the VTTs could be resected with the tumor conveniently in amputation cases, while complete thrombectomy was also recommended for limb‐salvage cases but this would be difficult. For type 3 cases, surgical resection of the VTT should be avoided in most occasions because of the extreme difficulties and risks and limited effects on local control, unless the patients respond extraordinarily well to neo‐adjuvant chemotherapy. For type 4 cases, surgical resection should be totally avoided. Sometimes ligation and resection of external iliac vein would not bring in serious occlusion of venous return due to existence of collaterals, but this is difficult to predict. Moreover, the effect of chemotherapy on VTT is generally minimal and the effect of radiotherapy remains unclear according to our experience.

### 4.6 Limitations

This study has several limitations. Firstly, the retrospective nature of this study might bring in recalling bias. However, all of the variables examined in this study were objective parameters such as demographic data, laboratory results, and radiological data, which could minimize the effects of the bias. Secondarily, we only included the patients with surgeries in a single center in this study that could result in selective bias. To our knowledge, this study has the largest case volume of pelvic osteosarcomas and VTTs from a single center in the literature, and thus the results would be valuable in this field. Moreover, only by pathological examination of the thrombus the diagnosis of VTT could be confirmed. Therefore, only cases with surgeries would be eligible for this study. Lastly, the interpretations about the predisposing factors of VTTs would need further investigations to verify. Although we proposed two correlated factors of VTT based on the clinical and radiological findings, the inclination of a tumor to form a VTT should be a complex issue resulted from anatomical, pathological and more importantly, genetic factors. The rationality of the classification and management of the VTTs would also need more investigations to validate.

## 5 CONCLUSIONS

The presence of VTT is not rare in patients with pelvic osteosarcoma, and its incidence is up to 15%. VTTs generally originate from the venous branches near the tumor and extend proximally within the lumen. Patients with tumors of chondroblastic subtype or with invasion of the L5/S1 intervertebral foramen might be of higher risk of developing VTTs. A comprehensive evaluation by multiple radiological methods including enhanced CT, MRI, and PET/CT could be helpful to identify VTTs before clinical decisions were made.

## CONFLICT OF INTEREST

The authors declared that there are no conflicts of interest.
